# Safety and effectiveness of CIMAvax-EGF administered in community polyclinics

**DOI:** 10.3389/fonc.2023.1287902

**Published:** 2024-01-18

**Authors:** Ramón A. Ortiz Carrodeguas, Geidy Lorenzo Monteagudo, Pedro P. Guerra Chaviano, Irene Álvarez Montané, Eva E. Salomón Saldívar, Leonardo Lobaina Lambert, Kirenia Camacho Sosa, Raúl Bermúdez Pino, Poncio Blanco Mustelier, Elba Valdés Rodríguez, Shairis González Piloto, Arelys Guerra de la Vega, Lizet Valdés Sánchez, Arasay Montes De Santis, Jenelly Parra Zabala, Carmen Viada González, Nadia Calvo Aguilera, Danay Saavedra Hernández, Orestes Santos Morales, Tania Crombet Ramos

**Affiliations:** ^1^ “Celestino Hernández Robau” Hospital, Medical Oncology Department. Santa Clara, Villa Clara, Cuba; ^2^ Center of Molecular Immunology, Clinical Research Direction, Havana, Cuba; ^3^ National Coordinating Center for Clinical Trials, Clinical Research Department, Havana, Cuba; ^4^ “María Curie” Hospital, Medical Oncology Department, Camagüey, Cuba; ^5^ “Joaquín Albarrán” Hospital, Medical Oncology Department, Havana, Cuba; ^6^ “Saturnino Lora” Hospital, Medical Oncology Department, Santiago de Cuba, Cuba; ^7^ “Faustino Pérez” Hospital, Medical Oncology Department, Matanzas, Cuba; ^8^ “Mario Gutiérrez Ardaya” Polyclinic, Family Medicine Department, Holguín, Cuba; ^9^ “José Luis Dubrocq” Polyclinic, Family Medicine Department, Matanzas, Cuba; ^10^ “Octavio de la Concepción y la Pedraja” Polyclinic, Family Medicine Department. Santa Clara, Villa Clara, Cuba; ^11^ “Camilo Cienfuegos” Polyclinic, Family Medicine Department, Artemisa, Cuba; ^12^ “Previsora” Polyclinic, Family Medicine Department, Camagüey, Cuba

**Keywords:** CIMAvax-EGF, community polyclinics, primary health care institutions, NSCLC, realworld-data (RWD)

## Abstract

In spite of the advances in immunotherapy and targeted therapies, lung cancer continues to be the leading cause of cancer-related death. The epidermal growth factor receptor is an established target for non-small cell lung cancer (NSCLC), and its overactivation by the ligands can induce accelerated proliferation, angiogenesis, and metastasis as well as proinflammatory or immunosuppressive signals. CIMAvax-EGF is an epidermal growth factor (EGF)-depleting immunotherapy that is approved for the treatment of NSCLC patients in Cuba. The study was designed as a phase IV trial to characterize the safety and effectiveness of CIMAvax-EGF in advanced NSCLC patients treated in 119 community polyclinics and 24 hospitals. CIMAvax-EGF treatment consisted of four bi-weekly doses followed by monthly boosters. Overall, 741 NSCLC patients ineligible for further cancer-specific treatment were enrolled. CIMAvax-EGF was safe, and the most common adverse events consisted of mild-to-moderate injection site reactions, fever, chills, tremors, and headache. For patients completing the loading doses, the median survival was 9.9 months. For individuals achieving at least stable disease to the frontline and completing vaccination induction, the median survival was 12 months. Most of the functional activities and symptoms evaluated through the European Organisation for Research and Treatment of Cancer (EORTC) QLQ-C30 questionnaire improved over time. In conclusion, this real-world trial demonstrated that CIMAvax-EGF was safe and effective in patients who were vaccinated in the maintenance scenario. A larger effect was seen in subjects with poor prognosis like those with squamous tumors and high EGF levels. Remarkably, this community-based intervention was very important because it demonstrated the feasibility of treating advanced lung cancer patients with active immunotherapy in primary care institutions. In addition to CIMAvax-EGF, patients received supportive care at the community clinic. Vaccine administration by the family doctors at the polyclinics reduced the patients’ burden on the medical oncology services that continued providing chemotherapy and other complex therapies. We conclude that community polyclinics constitute the optimal scenario for administering those cancer vaccines that are safe and require prolonged maintenance in patients with advanced cancer, despite the continuous deterioration of their general condition.

**Clinical trial registration:**

https://rpcec.sld.cu/trials/RPCEC00000205-En, identifier RPCEC00000205.

## Introduction

Lung cancer continues to be the leading cause of cancer-related mortality worldwide. It was estimated that 2,206,771 individuals were diagnosed with lung cancer in 2020 (crude rate of 28.3 cases per 100,000 inhabitants), which represents 11.4% of all types of cancer. Likewise, regarding mortality, 1,796,144 patients died from this disease, which represents 18% of deaths from all types of cancer (1).

Non-small cell lung cancer (NSCLC) is the most common type of lung cancer, accounting for approximately 85% of all lung cancer cases. Roughly 15% of patients with NSCLC are diagnosed in the early stages and present a survival greater than 50% at 5 years. However, more than 70% of cases are diagnosed in advanced stages, stage IIIB (loco-regional advanced disease) or stage IV (metastatic disease), when there are no options for curative treatment. In these cases, the curability rate is low, and close to 90% of patients die before 5 years (2).

Therapeutic options available to treat advanced lung cancer are chemotherapy, radiotherapy, targeted therapies, and immunotherapy. Molecular therapies targeting EGFR, ALK, ROS-1, and mutated KRAS, among others, are the standard of care for patients with sensitizing mutations (3, 4). For patients without mutations, immune checkpoint inhibitors in combination with platinum-based chemotherapy or as monotherapy have been established as the first-line treatment for metastatic disease (5, 6).

The epidermal growth factor receptor (EGFR) is a validated target for NSCLC (7, 8). The overactivation of the EGFR by its ligands can induce the malignant transformation of normal cells by inhibiting apoptosis or inducing cell proliferation, angiogenesis, metastasis, and proinflammatory or immunosuppressive signals (9, 10). The induction of epidermal growth factor (EGF) deprivation through active immunotherapy is an emerging concept developed by the Center of Molecular Immunology (Havana, Cuba). Our approach consists of manipulating the individual’s immune response to generate its own effector antibodies against the growth factor (11, 12). CIMAvax-EGF consists of a chemical conjugate including recombinant EGF and P64, another recombinant protein from *Neisseria meningitidis*. Montanide ISA 51 VG is used as adjuvant (13). The vaccine is intended to induce a humoral response against EGF, which can prevent the binding of the ligand to the receptor in the cancer cells, thus blocking the pro-tumoral signals (11, 14, 15).

Several clinical trials have been carried out in advanced NSCLC patients, with CIMAvax-EGF approved by the Cuban Regulatory Agency as maintenance after front-line therapy (14, 16–18). The present study was designed as a phase IV trial to characterize the safety and effectiveness of CIMAvax-EGF in IIIB/IV NSCLC patients treated in 119 community polyclinics (primary healthcare institutions) and 24 hospitals (secondary healthcare institutions) of Cuba. A secondary objective of the trial was to evaluate the survival benefit in relation to the EGF basal concentration as well as the quality of life.

## Materials and methods

Patients of any sex and age ≥ 18 years, with cytology or histology confirmation of NSCLC at the IIIB/IV stage, were enrolled. Other inclusion criteria were the following: individuals who had received the available cancer-specific treatment and had no other treatment option, subjects who signed the informed consent for the investigation, patients with Eastern Cooperative Oncology Group (ECOG) performance status of 0–3 (19) and life expectancy greater than 3 months. The main exclusion criteria comprised a previous history of hypersensitivity to compounds similar to the vaccine; pregnant, lactating, or post-partum women; and subjects with brain metastases.

The study did not have a pre-specified sample size but a safety hypothesis: the administration of CIMAvax-EGF to patients with advanced NSCLC will be safe given that the relative frequency of expected and unexpected serious adverse events, with a definite or probable causal relationship, will not exceed 1% of all patients. The study was opened for 3 years, during which those patients from the participating institutions who complied with the selection criteria and gave their informed consent were recruited.

CIMAvax-EGF was administered by the intramuscular route at a dose of 2.4 mg. The first four doses (induction phase) were administered every 14 days. Then, vaccination continued once a month as long as the patient’s conditions allowed, given that there were no safety concerns. Three days before the first vaccination, patients received an intravenous cyclophosphamide infusion at a low immunomodulatory dose (200 mg/m^2^). This treatment was administered on an outpatient basis in secondary healthcare institutions. The first CIMAvax-EGF dose was also administered at the hospitals so that trained oncologists could closely monitor the adverse events. Then, the rest of the vaccine doses were administered at the participating community polyclinics, according to the patient’s convenience. CIMAvax-EGF was administered at four injection sites in the two deltoid regions and gluteal regions.

Toxicities were graded according to the National Cancer Institute Common Toxicity Criteria, version 4.0. Lab tests included hematology (hemoglobin, complete blood count (CBC), and hematocrit) as well as alanine aminotransferase, aspartate aminotransferase, glycemia, bilirubin, alkaline phosphatase, creatinine, and urine analysis. These tests were repeated every 3 months. EGF concentration in serum at baseline was quantified with a validated enzyme immunoassay using an ultra-microanalytical system (20). In addition, survival from trial enrolment was evaluated. Several control variables including histology, ECOG performance status, response to front-line therapy, treatment compliance, and EGF concentration at baseline were evaluated in relation to survival. Quality of life was measured using the European Organisation for Research and Treatment of Cancer (EORTC) QLQ-C30 version 3 and the EORTC QLQ-LC13 questionnaires.

The protocol, case report forms, and informed consent were approved by centralized ethical committees (one per province), created *ad hoc* for this trial, to facilitate the approval process in a large number of primary (119) and secondary (24) healthcare sites. These centralized committees granted the conduction of the trial under the ethical principles embodied in the Declaration of Helsinki (21) and its subsequent updates (22). Since it was a phase IV trial with an approved drug, the national regulatory agency was notified. All patients provided signed informed consent. The trial was registered with the National Public Registry of Clinical Trials (https://rpcec.sld.cu/trials/RPCEC00000205-En).

All adverse events regardless of causality were reported. The frequency distributions of each type of event (related and unrelated) were estimated. Adverse events were also classified considering the intensity, causality, seriousness, treatment, and final results. Lab tests were evaluated considering the normal ranges defined by each clinical site. Overall survival was estimated in the intention to treat (ITT) and per-protocol population (patients who completed four induction doses) by using the standard Kaplan–Meier method. The association between other control variables and survival was evaluated using the standard Kaplan–Meier method and the log-rank test. Regarding quality of life, a longitudinal assessment was carried out every 3 months, and the data were interpreted according to the minimal relevant difference thresholds for the QLQ-C30 (23). Descriptive statistics were assessed for each of the scales before treatment and at months 3, 6, 9, and 12 from treatment. Differences with respect to baseline values for each measurement were estimated. Analyses were performed using SPSS-25.

## Results

In total, 741 subjects were included in the trial from January 2016 to December 2019. Last news data were available for all, except for one subject. At the time of follow-up termination and database lock (November 2021), 647 individuals had died, and 93 were alive. The inclusion took place in 24 hospitals nationwide, where the patients received cyclophosphamide and the first vaccine dose. Then, they were referred to 119 polyclinics according to the geographical location of their homes and the regionalization strategy established by each hospital. Before trial initiation, researchers from the polyclinics including the family doctors, nurses, pharmacists, and site coordinators were trained in good clinical practices and received courses on lung cancer, immunotherapy, and cancer vaccines, particularly on CIMAvax-EGF mechanism of action, safety, and efficacy. In addition, family doctors and nurses were qualified in CIMAvax-EGF preparation and mode of administration. Patient demographic and tumor characteristics are described in **Table 1**. The majority were male, older than 60 years, with stage IV NSCLC, and were current or former smokers. There was a similar proportion of subjects with squamous or adenocarcinomas, and a large percentage (87%) had previous chemotherapy with stable or progressive disease as the best response.

**Table 1:** Patient demography and baseline characteristics.

**Age**	≤60 years	188 (25.5%)
	>60 years	553 (74.6%)
	Mean (range)	65.22 (23–94)
**Gender**	Male	451 (60.9%)
	Female	290 (39.1%)
**Ethnic origin**	White	513 (69.2%)
	Mixed	1 45 (19.6%)
	Black	83 (11.2%)
**Disease stage**	IIIB	256 (34.5%)
	IV	485 (65.5%)
**ECOG PS**	0	305 (41.2%)
	1	247 (33.3%)
	2	138 (18.6%)
	3	51 (6.9%)
**Smoking status**	Current smoker	241 (32.%)
	Former smoker	405 (54.7%)
	Never smoker	95 (12.8%)
**Histology**	Squamous cell carcinoma	258 (34.8%)
	Adenocarcinoma	242 (32.7%)
	Large cell carcinoma	135 (18.2%)
	NSCLC NOS	97 (13.1%)
	Other	9 (1.1%)
**First-line treatment**	Chemotherapy	647 (87.3%)
	Radiotherapy	163 (22%)
	Not received	94 (12.6%)
**Response to first-line**	Complete response	24 (3.2%)
	Partial response	180 (24.3%)
	Stable disease	208 (28.1%)
	Progression disease	208 (28.1%)
	NA	121 (16.3%)
**[EGF] pg/ml**	<870 pg/ml	442 (59. 6%)
	≥870 pg/ml	225 (30.5%)
	NA	74 (9.9%)

ITT population (n = 741). NOS, not otherwise specified; NA, not available; ECOG PS, Eastern Cooperative Oncology Group performance status; NSCLC, non-small cell lung cancer; EGF, epidermal growth factor.

The CIMAvax-EGF scheme consisted of four induction and monthly maintenance doses. Overall, 544 (73.4%) individuals completed induction: 77 (10.4%) only received the first four doses, 328 (44.2%) had between five and 15 doses, 78 patients (10.5%) had 16 to 26 CIMAvax-EGF administrations, and 61 subjects (8.32%) received more than 27 injections.

Nineteen patients (2.4%) did not receive any vaccine dose on account of rapid deterioration, while 178 (24%) could not complete the induction scheme of four doses. The most frequent cause of definitive treatment interruption at any time was death (280 patients, 41.2%), followed by worsening of the patient’s condition (207 patients, 27.9%). Death or performance status deterioration was not attributed to CIMAvax-EGF but to the natural course of the disease.

For the safety analysis, 722 patients who received at least one immunization with CIMAvax-EGF were considered. Among these individuals, 459 (57.29%) had at least one adverse event. Overall, 2,389 adverse events were reported. Of these, the largest number (1,846 events) occurred between doses 1 and 6. The most frequent adverse events regardless of causality were injection site pain (268; 11.2%), dyspnea (163; 6.8%), fever (136; 5.7%), chills (83; 3.5%), headache (81; 3.4%), and nausea (80; 3.3%). Among the 2,389 events, 993 (41.6%) were classified as definitively, probably, or possibly related to CIMAvax-EGF. Concerning related adverse events, 667 (67.1%) were mild, 269 (27.0%) were moderate, and 27 (2.7%) were severe. The most common related adverse events included injection site pain, fever, chills, tremors, headache, and nausea. Three patients (0.4%) had five serious related events consisting of anaphylactic shock, tremors (two events), redness of the upper limbs, vagal reaction, and chest pain. The longitudinal evaluation of the most important hematology and biochemistry tests during the first year of CIMAvax-EGF administration is shown in **Table 2**.

**Table 2:** Blood test results before and after CIMAvax-EGF treatment for the first year.

Laboratory parameter	Baseline	Month 3	Month 6	Month 9	Month 12
Hemoglobin (g/L) Mean (95% CI)	116.8 (15)	123.6 (15)	121.2 (16)	118.2 (17)	118.8 (11)
ALC (10^9^/L) Mean (SD)	8.7 (3)	8.7 (2)	8.6 (2)	9.2 (3)	8.0 (2)
Neutrophil % Mean (SD)	65.6 (12)	64.7 (10)	63.9 (13)	62.2 (13)	61.8 (10)
Lymphocyte % Mean (SD)	29.8 (11)	28.5 (10)	31.4 (12)	30.9 (11)	30.5 (12)
Hematocrit Mean (SD)	36.3 (4)	38.3 (4)	37.6 (4)	36.4 (5)	37.2 (4)
Platelets (10^9^/L) Mean (SD)	265.7 (114)	244.2 (90)	267.7 (101)	248.3 (55)	219.2 (46)
AST (UI/L) Mean (SD)	23.1 (17)	23.5 (24)	22.0 (10)	25.9 (15)	25.5 (18)
ALT (UI/L) Mean (SD)	20.7 (17)	22.0 (23)	21.6 (19)	23.2 (22)	23.7 (21)
Glycemia (mmol/L) Mean (SD)	5.6 (1)	5.6 (1)	5.7 (1)	6.3 (2)	5.8 (1)
Bilirubin (μmol/L) Mean (SD)	9.8 (6)	10.1 (5)	9.8 (4)	9.9 (7)	9.8 (4)
ALP (UI/L) Mean (SD)	240.0 (156)	246.4 (202)	235.1 (101)	201.6 (131)	145.6 (75)
Creatinine (μmol/L) Mean (SD)	91.4 (32)	95.4 (28)	96.1 (31)	105.5 (32)	103.3 (30)

ALC, absolute leucocyte count; AST, aspartate aminotransferase; ALT, alanine aminotransferase; ALP, alkaline phosphatase.

Survival was analyzed in the ITT scenario and in those patients who completed vaccination induction. The mean and median survival time (ST) for all patients irrespective of treatment compliance was 13.9 (95% CI 12.6–15.3) and 7.0 months (95% CI 6.3–7.8). The 12- and 24-month survival rates were 32% and 14.7%. For the 550 patients who received at least four vaccine doses, the mean and median STs were 17.6 (95% CI 15.9–19.3) and 9.9 months (95% CI 8.8–11), respectively. The 12- and 24-month survival rates were 42% and 19.5%, respectively.

Finally, survival was evaluated in the subgroup of patients who completed front-line therapy for the advanced disease, achieved at least stable disease (maintenance scenario), and received the four loading doses. In our data set, 412 (55.6%) individuals received front-line therapy, reaching complete, partial, or disease stabilization, and 328 (79.6%) completed induction vaccination. For this subpopulation, the mean and median survival were 20.4 months (95% CI 18–22.8) and 12 months (95% CI 10.6–13.4), respectively. Survival rates were 40.9% and 19.8% after 1 and 2 years, respectively.

In patients who were classified as unfit for chemotherapy and those in progressive disease upon front line, the median survival was 6.9 and 7.4 months, respectively, if completed induction.

**Figure 1:**
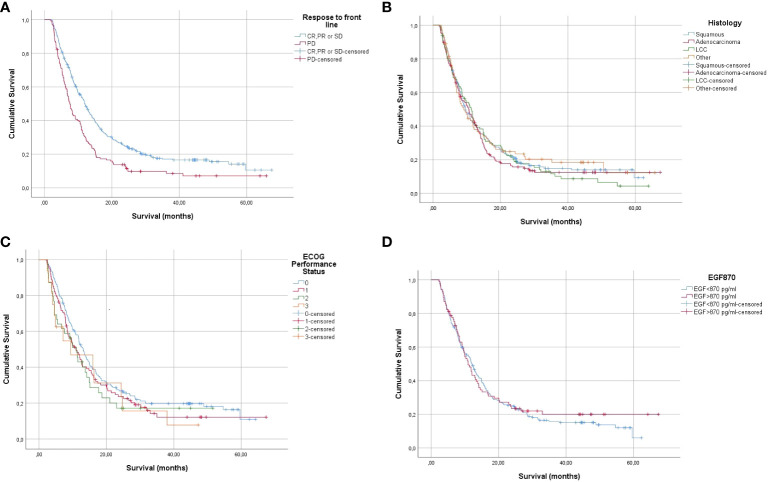
Kaplan–Meier estimates of overall survival of NSCLC patients completing loading CIMAvax-EGF doses according to response to front-line chemotherapy **(A)**, histology **(B)**, ECOG PS **(C)**, and EGF concentration at baseline **(D)**. CR, complete response; PR, partial response; SD, stable disease; PD, progressive disease; NSCLC, non-small cell lung cancer; ECOG PS, Eastern Cooperative Oncology Group performance status; EGF, epidermal growth factor.

In the per-protocol scenario, a separate survival analysis was carried out for some of the control variables including response to front-line chemotherapy, histology, ECOG PS, and EGF concentration at baseline. **Figure 1** illustrates the survival curves.

As expected, significant survival differences were detected for patients achieving at least stable disease after chemotherapy (log-rank p = 0.00011). The median survival of individuals with complete, partial, or stable disease before CIMAvax-EGF was 12 months vs. 7.4 months for those patients who progressed to front-line chemotherapy. Notably, no survival differences were seen in individuals with distinct ECOG performance status, different cancer histology, and EGF concentrations above or below 870 pg/ml. The mean and median survival for subjects with squamous tumors were 20.7 and 11.6 months vs. 21.34 and 13.13 months, respectively, for the adenocarcinoma histology. The 1- and 2-year survival rates were 47.9% and 26.5% in the squamous carcinoma patients and 56.6% and 22.6% in the adenocarcinoma subset. The median survival of the patients with baseline EGF concentration lower or higher than 870 pg/ml was 12.1 and 10.9 months. The survival rate at 24 months was 25.5% and 23.4% for the referred groups.

The evaluation of the antitumor response was not an objective of this phase IV trial since it has been characterized in previous studies (12, 16, 24, 25). Overall, across several CIMAvax-EGF studies, the disease control rate ranges from 30% to 40% and consists mainly of stable disease (12, 16, 24, 25). Notably, in phase 1 where CIMAvax-EGF was combined with nivolumab after the progression of the disease (second-line setting), 33% of the patients achieved partial response, while the overall disease control rate was 50% (26).

**Figure 2:**
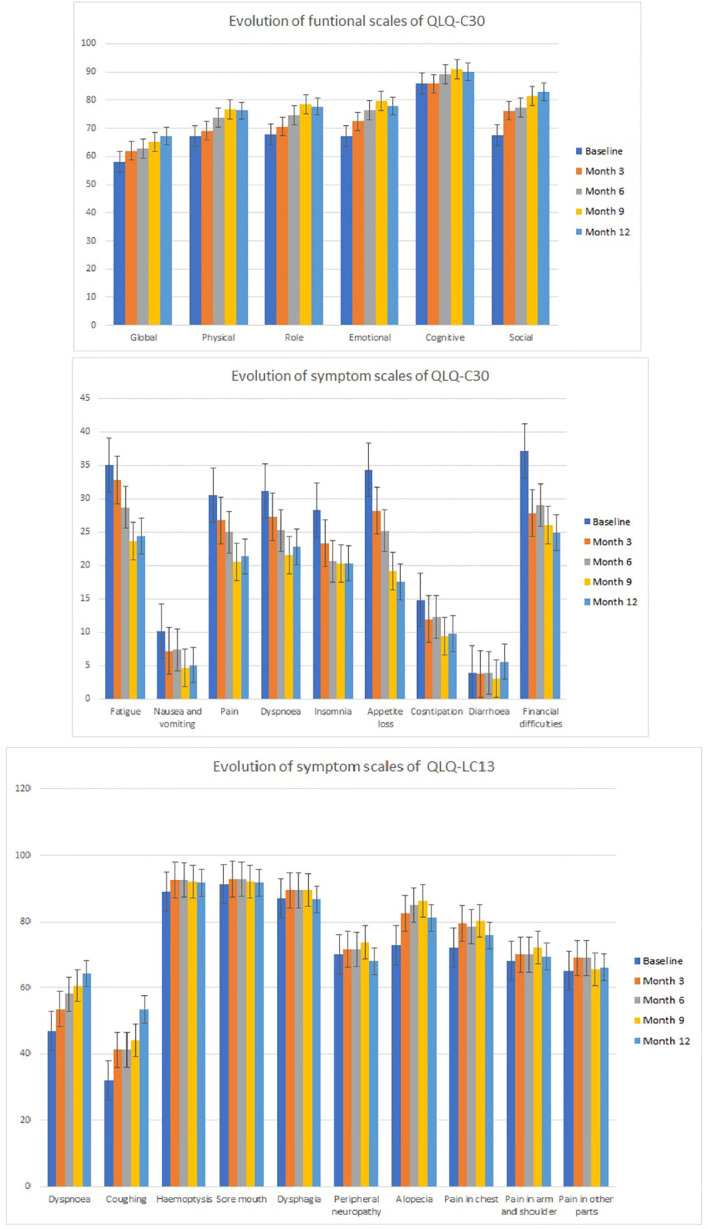
Evaluation of the functional and symptom scales of the EORTC QLQ-C30 questionnaire and the symptoms scale from the EORTC QLQ-L13 questionnaire. EORTC, European Organisation for Research and Treatment of Cancer.

The quality of life (QoL) analyses were performed for all the subjects who completed the questionnaires (QLQ-C30 and QLQ-L13) at different time intervals. At baseline, information was available for 665 patients (89.7%), while at month 12, 101 out of the 228 (44.2%) individuals who were alive provided QoL information. **Figure 2** presents the results of the functional scales of the QLQ-C30 as well as the symptom scales from the general and lung questionnaires. Each of the items was evaluated over time, and the scoring was compared to the baseline. A difference of 5 to 10 points was classified as a small effect, while a change of 10 to 20 points was cataloged as a moderate effect (27, 28). Overall, the global quality of life, as well as the physical, role, emotional, social, and cognitive functions, improved over time. Regarding the functional scales, after 12 months (the last available evaluation), there was a small improvement in the global and physical functions. Likewise, there was a moderate improvement in the role, emotional, and social functions.

Concerning the symptom evaluation of the QLQ-C30, all items became better with time except diarrhea. A minor recuperation was seen in nausea, pain, dyspnea, insomnia, and constipation, while a moderate improvement in fatigue, appetite loss, and financial difficulties was observed in month 12.

Finally, the QLQ-LC-13 survey detected an improvement in the dysphagia and neuropathy in addition to a small worsening (less than 5 points) in the hemoptysis as well as in the sore mouth, chest, and arm/shoulder and pain in other parts. The magnitude of the worsening had no clinical relevance and can be interpreted as a stabilization of the referred symptoms. On the contrary, in spite of vaccination, there was a moderate worsening of dyspnea and cough. These symptoms were associated with the underlying lung cancer condition and not with CIMAvax-EGF.

## Discussion

This was a phase IV clinical trial of CIMAvax-EGF in patients with advanced NSCLC who did not have any further therapeutic alternative according to the national guidelines. Patients were recruited by the hospitals’ oncologists, who also administered low-dose cyclophosphamide and the first vaccine doses. Then, individuals continued vaccination at the primary care institutions. Clinical evaluation was carried out by the oncologists together with the family physicians every 3 months.

The trial demonstrated that CIMAvax-EGF administration was feasible at the community polyclinics. CIMAvax-EGF is an EGF-depleting immunotherapy with a very good safety profile, which requires monthly maintenance doses that can be prolonged according to the individual response and performance status. In our data set, roughly 20% of the patients were vaccinated over 1 year or more, and remarkably, at the moment of this writing, 44 patients remained vaccinated after 3 years or more. The vaccinated subjects and caregivers acknowledged the convenience of the regular vaccine administration near their houses.

Vaccine compliance was good, and 73% of the patients completed the vaccine loading period. In the previous phase III trial at the hospitals, 81% of the patients successfully finished induction (18). Apart from the referred registration study where all the patients had had front-line platinum doublets and only 5% had progressive disease at enrolment (18), in this real-world scenario trial, 44.4% were in progression or did not have any previous therapy (unfit for chemotherapy).

CIMAvax-EGF was very well tolerated, and the trial fulfilled its safety hypothesis since less than 1% of the patients had serious related adverse events. The most frequent adverse events were consistent with those reported in the previous studies (11, 12, 15, 17, 18). CIMAvax-EGF safety profile was very good in comparison to other drugs used in the maintenance or second-line treatment of advanced NSCLC. In the case of docetaxel, hematological toxicity such as neutropenia, anemia, and thrombocytopenia, as well as non-hematological toxicity including diarrhea, nausea, and vomiting, have been reported (29). Pemetrexed suppresses bone marrow function, but patients can also develop skin reactions and serious renal events, including acute renal failure (30). Alternatively, immune checkpoint inhibitors that boost the natural immune response may induce immune-related adverse events like pneumonitis, colitis, hepatitis, and hypothyroidism (31, 32).

CIMAvax-EGF is being administered to advanced lung cancer patients who are immunocompromised on account of their older age, the cancer itself, and the previous platinum-based chemotherapy, in most cases (33). Apart from conventional chemotherapy that exerts its action directly, this EGF-depleting immunotherapy needs to break the tolerance against a self-growth factor. In consequence, administering a minimum number of doses (four loading doses) is needed to trigger a protective immune response that may slow the progression of the disease. According to our previous data, before completing induction, the frequency of patients achieving a good response against EGF (antibody titles >1:4,000) is less than 50% (14, 34). Our team has consistently found that repeated immunization is also correlated with a higher capacity of blocking the EGF/EGFR binding and inhibiting the EGFR phosphorylation. Furthermore, there is a significant correlation between a good antibody response and survival (11, 14, 34).

This was a phase IV trial carried out in the conditions of routine medical practice. Overall, 27% of the patients did not complete the initial four doses, including 19 individuals who were not vaccinated at all on account of rapid worsening. These figures highlight the importance of selecting the right patients for an active immunotherapy like CIMAvax-EGF. Patients with poor performance status who progressed or were deemed unfit for platinum-based chemotherapy are not the best candidates for vaccine monotherapy.

However, CIMAvax-EGF has been safely administered to stage IV NSCLC patients with progressive disease, in combination with nivolumab in a phase I/II trial, at the Roswell Park Comprehensive Cancer Center (Buffalo, NY). For all patients receiving the anti-PD1 antibody plus the vaccine, the median survival was 13.5 months, while for individuals completing CIMAvax-EGF induction, the median survival was 18.3 months. Particularly, patients with KRAS wild type had a very high median survival (21.7 months) (26). Since KRAS mutations predict resistance to the EGF/EGFR blockade, it is rational to anticipate a larger benefit of CIMAvax-EGF in patients with KRAS wild-type tumors. The same concept has been validated in colorectal cancer individuals treated with the anti-EGFR antibodies cetuximab or panitumumab (35, 36).

In our data set, patients who completed the induction after at least disease stabilization to front-line therapy had a median survival of 12 months. This is comparable with the survival achieved with other drugs used as switch maintenance—docetaxel (12.3 months) (37), pemetrexed (13.4 months) (38), and erlotinib (12.0 months) (39)—but with much lower toxicity. Nevertheless, the preferred approach in current medical practices is to use continuation maintenance with one or some of the front-line drugs for the advanced stage, comprising immune checkpoint inhibitors. The nature of the survival estimation in the continuation maintenance scenario precludes any comparison with switch maintenance, where survival is estimated from the completion of the front line.

Notably, there were no significant differences in the survival of patients with squamous or non-squamous tumors. This result suggests that subjects with squamous histology had greater benefits after vaccination since it is well accepted that the prognosis of adenocarcinoma patients is much better (40, 41). Previous randomized clinical trials have also found a bigger effect in patients with squamous carcinomas, presumably associated with the greater expression of unmutated EGFR in this tumor type (42).

Likewise, patients with low or high EGF concentration in serum had non-different survival. This is precisely what is expected. EGF concentration after front-line therapy in NSCLC has been proposed as a poor prognostic biomarker of the disease and also as a predictive biomarker of higher CIMAvax-EGF efficacy (18, 42). It means that patients with low EGF have a better prognosis, while patients with high EGF would have a poorer prognosis but a larger survival gain with CIMAvax-EGF.

Quality of life is very important since the majority of conventional antitumor treatment for lung cancer increases survival at a cost of significant toxicity and QoL deterioration (43, 44). Globally, there was a trend toward improvement over time in the functional activities and the symptoms evaluated through the general questionnaire. However, symptoms particularly associated with lung cancer evidenced small deterioration lacking clinical relevance except the dyspnea and coughing that became worse after 6 months, coinciding with the natural history of the disease. We concluded that CIMAvax-EGF did not negatively affect quality of life, but on the contrary, it improved some of the functions and symptoms of late-stage cancer patients. Major limitations of this assessment are associated with the lack of a control arm that prevents the evaluation of the symptoms and functional scales in untreated individuals and with the missing information, mainly at late time points.

In summary, this clinical trial was particularly important because it demonstrated the feasibility and advantages of treating advanced lung cancer patients with active specific immunotherapy in primary healthcare institutions. The use of CIMAvax-EGF was extended to a large number of community polyclinics for the first time. CIMAvax-EGF administration by the family doctors at the community polyclinics significantly reduced the patients’ burden on the medical oncology services that continued providing chemotherapy and other complex therapies. Family doctors also provided supportive therapy as well as end-of-life care. This real-world scenario study confirmed that CIMAvax-EGF, as monotherapy, was safe and effective in patients who were vaccinated in the maintenance setting after a good response to the front-line therapy. The importance of vaccinating the right patients with an adequate life expectancy and performance status was also confirmed. Preliminary findings also support the largest impact of CIMAvax-EGF in patients with poor prognosis like those with squamous tumors and high EGF serum levels. New clinical trials where CIMAvax-EGF is combined with other immunomodulatory drugs including anti-PD1 antibodies are ongoing in NSCLC patients.

## Data availability statement

The raw data supporting the conclusions of this article will be made available by the authors, without undue reservation.

## Ethics statement

The studies involving humans were approved by Celestino Hernández Hospital ethic committee and other IRBs established at hoc in each province for the clinical trial. The studies were conducted in accordance with the local legislation and institutional requirements. The participants provided their written informed consent to participate in this study.

## Author contributions

ROC: Conceptualization, Formal Analysis, Investigation, Supervision, Writing – review & editing. GLM: Conceptualization, Formal Analysis, Investigation, Supervision, Writing – review & editing. PGC: Investigation, Methodology, Supervision, Writing – review & editing. IÁM: Investigation, Writing – review & editing. ESS: Investigation, Writing – review & editing. LLL: Investigation, Writing – review & editing. KCS: Investigation, Writing – review & editing. RBP: Investigation, Writing – review & editing. PBM: Investigation, Writing – review & editing. EVR: Investigation, Writing – review & editing. SGP: Investigation, Writing – review & editing. AGV: Investigation, Writing – review & editing. LSV: Data curation, Formal Analysis, Investigation, Methodology, Writing – review & editing. AMS: Data curation, Software, Writing – review & editing. JPZ: Data curation, Writing – review & editing. CVG: Data curation, Formal Analysis, Writing – review & editing. NCA: Data curation, Writing – review & editing. DSH: Investigation, Supervision, Writing – review & editing. OSM: Investigation, Project administration, Supervision, Writing – review & editing. TCR: Conceptualization, Formal Analysis, Investigation, Project administration, Supervision, Writing – original draft, Writing – review & editing.
